# Does Levetiracetam Administration Prevent Cardiac Damage in Adulthood Rats Following Neonatal Hypoxia/Ischemia-Induced Brain Injury?

**DOI:** 10.3390/medicina54020012

**Published:** 2018-04-10

**Authors:** Serkan Gurgul, Belgin Buyukakilli, Mustafa Komur, Cetin Okuyaz, Ebru Balli, Tuba Ozcan

**Affiliations:** 1 Department of Biophysics, Faculty of Medicine, Gaziantep University, TR-27310 Gaziantep, Turkey; 2 Department of Biophysics, Faculty of Medicine, Mersin University, TR-33343 Mersin, Turkey; bbuyukakilli@yahoo.com; 3 Department of Child Health and Disease, Faculty of Medicine, Mersin University, TR-33343 Mersin, Turkey; drmustafakomur@yahoo.com (M.K.); okuyazc@mersin.edu.tr (C.O.); 4 Department of Histology and Embryology, Faculty of Medicine, Mersin University, TR-33343 Mersin, Turkey; ebruballi@mersin.edu.tr; 5 Department of Histology and Embryology, Faculty of Medicine, K. Sütcü Imam University, TR-46040 Kahramanmaraş, Turkey; tuba84_ozcan@hotmail.com

**Keywords:** cardiac mechanical performance, heart ultrastructure, hypoxia, ischemia, levetiracetam

## Abstract

Cardiovascular abnormalities are widespread when a newborn is exposed to a hypoxic-ischemic injury in the neonatal period. Although the neuroprotective effects of levetiracetam (LEV) have been reported after hypoxia, the cardioprotective effects of LEV have not been documented. Therefore, we aimed to investigate whether levetiracetam (LEV) has a protective effect on cardiac-contractility and ultrastructure of heart muscle in rats exposed to hypoxia-ischemia (HI) during the neonatal period. A total of 49 seven-day-old rat pups were separated into four groups. For HI induction, a combination of right common carotid artery ligation with 8% oxygen in seven-day-old rat pups for 2 h was performed for saline, LEV100, and LEV200 groups. Just after hypoxia, LEV100 and LEV200 groups were administered with 100 mg/kg and 200 mg/kg of LEV, respectively. The arteries of rats in the control group were only detected; no ligation or hypoxia was performed. At the end of the 16th week after HI, cardiac mechanograms were recorded, and samples of tissue were explored by electronmicroscopy.While ventricular contractility in the control group was similar to LEV100, there were significant decreases in both saline and LEV200 groups (*p* < 0.05). Although ventricular contractile duration of the control and saline groups was found to be similar, durations in the LEV100 and LEV200 groups were significantly higher (*p* < 0.05). After HI, mitochondrial damage and ultrastructural deteriorative alterations in ventricles and atriums of the LEV-administered groups were significantly less severe than the saline group. The present study showed that neonatal HI caused long-term cardiac dysfunction and ultrastructural deteriorations in cardiac muscles. LEV administration just after HI might possess some protective effects against myocardial damage and contractility.

## 1. Introduction

Hypoxic-ischemic brain injury (HIBI) is one of the major reasons of neonatal death and neurological and neuropsychological defects, including mental retardation, cerebral palsy, epilepsy, and learning disability, that are widely seen in the neonatal period worldwide [1]. As a consequence of exposure to hypoxia-ischemia (HI) in the neonatal term, multiple biochemical mechanisms and pathways are also shown to contribute to the progression of multi-organ damage, including cardiovascular abnormalities [2]. The incidence varies from 29 to 78%, and long-term follow-up of these infants is crucial because of the risk of developing late-onset cardiovascular disease [2,3,4,5,6]. The oxygen deprivation secondary to a hypoxic-ischemic injury (HII) is thought to cause myocardial damage, which develops a low cardiac output and reduced myocardial contractility [2,7,8,9,10]. Despite the considerable progression in the understanding of the underlying mechanisms of HII, including energy failure, membrane depolarization, the production of oxygen–free radicals, and lipid peroxidation [11,12,13], an efficacious treatment plan or strategy is still not available [14].

Racetams are a group of synthetic nootropics or cognitive enhancing drugs, such as piracetam, phenylpiracetam, and pramiracetam, which share a pyrrolidone ring. Although levetiracetam (LEV), seletracetam, and brivaracetam have similar structural features with the other members of this drug class, they demonstrate anti-epileptic activity [15]. LEV [(−)-(S)-α-ethyl-2-oxo-1-pyrrolidine acetamide] is broadly prescribed by clinicians for treating epileptic seizures because it has a large spectrum of anti-epileptic activity against different seizure types and syndromes with a good efficacy and safety profile [16,17,18]. Unlike seletracetam and brivaracetam, LEV was approved by the FDA in 1999 as an add-on treatment for partial-onset seizures in adults and children (≥4 years of age), for adults and adolescents with myoclonic epilepsy, and most recently (in 2012) for partial onset seizures in infants (≥1 month of age) [15,17]. Brivaracetam also gained FDA approval in 2016 for use in adults with partial onset seizures. However, it is not approved for pediatric patients (≤18 years of age) with epilepsy. Promising data obtained from recent studies regarding the efficacy of LEV in neonates and recent surveys on the use of the anti-epileptic drugs in neonatal units indicate that LEV is frequently used as second-line treatment [17]. Besides the considerable anti-seizure and anti-epileptic features, LEV also has good efficacy and tolerability in the management of movement and mood disorders, such as dyskinesia, myoclonus, generalized dystonia, and refractory bipolar disorder [16,19,20,21,22]. Several experimental studies have been performed to evaluate the neuroprotective effects of LEV on neonatal HIBI using histopathological, biochemical, and behavioral test methods [1,23,24,25,26]. Besides the anti-inflammatory, anti-oxidative, and anti-apoptotic effects of LEV, elevated learning abilities have been also reported after LEV administration [1,16,23,26,27,28]. Moreover, muscarinic acetylcholine receptor activity has been shown to be increased after LEV administration in the pilocarpine-induced seizure model [29]. The receptors mediate various physiological functions, including heart rate and force, smooth muscle contraction, and neurotransmitter release [30]. Based on this evidence, LEV is a candidate drug for the prevention of neonatal HII-induced multiple organ dysfunction and neurological and/or neuropsychological defects.

In the present study, as oxidative stress, apoptosis, and inflammation may play a role in the pathogenesis of neonatal HII [1,31,32], we hypothesized that LEV can be useful in the prevention of neonatal HII-associated cardiac dysfunction and myocardial damage in adulthood. Thus, we aimed to investigate whether LEV possesses a protective effect on cardiac contractility and ultrastructure of cardiac muscle of sixteen-week rats exposed to HI during the neonatal period (seventh day after birth). To the best of our knowledge, this is the first paper investigating the effects of LEV on cardiac mechanical performance and ultrastructural properties of cardiac muscle in adulthood against neonatal HII.

## 2. Experimental Section

### 2.1. Animal Preparation and Surgical Procedure

A total of 49 seven-day-old Wistar-Albino male rat pups, 11.82 ± 0.05 g, were used in this study. The rats were purchased from Clinical and Experimental Research Laboratory of Mersin University. All experiments and protocols described in the present study were performed in accordance with the guidelines of the European Convention for the Protection of Vertebrate Animals Used for Experimental and Other Scientific Purposes and also approved by the Medical Faculty Experimentation Ethics Committee of Mersin University.

The rats were divided randomly into four groups as follows:

1. The control group (*N* = 7): After a median neck incision, carotid arteries of the rats in this group were detected without ligation. No hypoxia was performed, and no medication was given.
2. The saline treated group (*N* = 14): 0.2 mL of saline was injected intraperitoneally immediately after hypoxia. One rat in this group died during the surgical procedure.
3. The 100 mg/kg Levetiracetam treated group (LEV100; *N* = 14): The rat pups were intraperitoneally administered with 100 mg/kg of LEV dissolved in saline immediately after hypoxia. Three rats in this group died during the surgical procedure (one rat) and the hypoxia period (two rats).
4. The 200 mg/kg Levetiracetam treated group (LEV200; *N* = 14): The rat pups were intraperitoneally administered with 200 mg/kg of LEV dissolved in saline immediately after hypoxia. Five rats in this group died during the surgical procedure (two rats) and the hypoxia period (three rats).

In the literature, experimental studies performed in rodents have demonstrated that the LEV has a high therapeutic index (TI; >148) [33]. Studies also have shown that a long-term oral treatment up to doses of 1800 mg/kg/day in neonatal and juvenile rats do not cause deaths, organ failure, or other irreversible toxicity [34]. In addition, NOAEL (no observed adverse effect level) was found for 200 mg/kg of LEV in rabbit fetus for the embryo-fetal development. Manthley and colleagues demonstrated that LEV possessed a lack of neurotoxic effects at all studied doses (5, 10, 25, 50, and 100 mg/kg per dose, similar to doses in humans) in seven-day-old rats [27]. Human studies have demonstrated that LEV was well tolerated and very effective at 120 mg/kg/day, indicating that higher doses may be well tolerated in some children [35]. In our previous study, single administration of 100 mg/kg and 200 mg/kg of LEV just after the HII showed a significant neuroprotective effect, which was demonstrated by the results of late-period behavioral experiments at the 16th week of the study [1]. On the basis of that knowledge, we have about the LEV, 100 mg/kg and 200 mg/kg of LEV doses were chosen for the present study.

In the present study, all animal procedures applied to induce HIBI were carried out in accordance with the Levine-Rice model [36]. By far the most commonly used animal for models of neonatal HIBI is the seven-day-old rat pup [37]. We used a combination of right common carotid artery ligation with 8% oxygen in seven-day-old rat pups for 2 h, as described in Rice et al. [36], Komur et al. [1], and Buyukakilli et al. [38]. After the HI induction, the rats were housed under standard conditions (12 h light/dark cycle, 22 ± 2 °C, relative humidity 50–70%, standard rat nutrients, and purified drinking water ad libitum) till the sixteenth week.

### 2.2. Cardiac Mechanography

At the end of the 16th week following hypoxia-ischemia, body weights (BW; g) of each rat were measured just before the cardiac mechanogram recordings of the heart. All of the mechanogram recordings in the present study were made in-situ as described in detail by Buyukakilli et al. [38]. After the mechanogram recordings, the study was terminated. The heart of each animal was harvested without distorting its integrity, weighed (heart weight (HW; g)) via an analytical balance (Sartorius TE214S, Data Weighing Systems Inc., Elk Grove, IL, USA), and was used for the electron microscopic evaluation. HW to BW ratio (HW/BW; mg/g) was calculated using data collected from HW and BW.

### 2.3. Sample Preparation for Electron Microscopy

For transmission electron microscopic (TEM) evaluation of the heart tissue, samples were fixed with 2.5% gluteraldehyde, postfixed with 1% osmium tetroxide, dehydrated in graded alcohol series, cleared with propylene oxide, and embedded in epon. Thin sections (50–70 nm) were cut by an ultramicrotome (Leica UCT-125, Leica Microsystems GmbH, Wetzlar, DE, USA) and contrasted with uranyl acetate and lead citrate. Sections were examined and photographed under a transmission electron microscope (JEOL JEM-1011, JEOL Ltd., Tokyo, Japan).

### 2.4. Ultrastructural Analysis of Mitochondria

Electron micrographs were taken with a magnification of 10.000×, indiscriminately, from atrium and ventricle for all four groups. A total of 10 different fields for each biopsy and 20 mitochondria in every field were evaluated. In total, 200 mitochondria per sample were graded. Images were analyzed by one experienced investigator unaware of the sequence of sampling. Mitochondrial damage was scored by appointing a numerical value from 0 to 3 to each mitochondrion. This scoring system modified from the score that was previously used by Milei et al. [39]. Grading scale was described as follows: 0—normal, 1—initial swelling (cristae fragmentation, diminished matrix density), 2—more evident swelling than in grade 1 and architectural deterioration, 3—findings as in grade 2 plus rupture of inner and outer mitochondrial membranes. The obtained mean was expressed for each grade as a percentage of the total count of mitochondria enumerated per sample.

### 2.5. Statistical Analysis

The Shapiro-Wilk test was performed on all data to determine the normality distribution of the data. If the distribution of normality was met, the data were expressed as mean and standard deviation (SD) and were analyzed by one-way analysis of variance (ANOVA) followed by Tukey’s HSD or Games-Howell multiple comparison tests. If not, the data were expressed as median (interquartile range), and Kruskal-Wallis or Welch post-hoc tests were used for the comparisons. The difference (two-tailed *p*) of less than 0.05 was regarded as significant. All statistical analyses were performed using the statistical package program SPSS for Windows (Release 11.5, Lead Technologies Inc., Chicago, IL, USA) and Statistica 8.0 Demo Program (StatSoft Inc., Tulsa, OK, USA) as applicable.

## 3. Results

### 3.1. Body Weight, Heart Weight, and Heart to Body Weight Ratio Results of the Groups

BW, HW, and HW/BW ratios of all groups of rats are listed in Table 1. As expected, the mean values of BW in the saline group were significantly reduced when compared to those of the control group (*p* < 0.05). The highest BW mean was detected in the control and LEV200 groups with no significant differences among them. The differences regarding BW between the control and both saline and the LEV100 groups were found to be statistically significant (*p* < 0.05 for both comparisons). The highest averages in HW and HW/BW ratio were detected in the control and levetiracetam-treated groups (LEV100 and LEV200) with no significant differences among them. However, the difference in HW between the control and the saline groups was found statistically significant (*p* < 0.05). No other significant differences were determined among the groups with regard to the tested parameters.

### 3.2. Cardiac Mechanogram Results of the Groups

Table 2 lists the atrial contractile force and duration and ventricular contractile force and duration values of the rats for each group. No statistically significant differences were found among the groups with regard to the atrial contractile force. Although the atrial contractile duration values of the control, the saline, and the LEV200 rats were found to be similar, the atrial contractile duration of LEV100 was significantly higher than that of the control (*p* < 0.01), the saline (*p* < 0.01), and the LEV200 (*p* < 0.05) groups. The highest ventricular contractile force values were determined from the control and the LEV100 groups with no significant difference among them. The difference between the control and the saline groups in ventricular contractile force was found significant (*p* < 0.01), indicating that the HII may cause ventricular dysfunction in adulthood rats. Although the decrease in ventricular contractile force in LEV200 was found significant in comparison to that of both control and the LEV100 groups (*p* < 0.01), the ventricular contractility in control and LEV100 groups were found similar. The lowest ventricular contractile duration was obtained from the control group. The ventricular contractile duration of the saline rats was higher than that of the control group, but the difference was found to be not significant. The ventricular contractile duration of LEV100 and LEV200 rats were significantly higher than that of the control and saline groups (*p* < 0.001, respectively). No other significant differences were found among the groups with respect to the force or duration of atrial and ventricular contractility.

### 3.3. Electron Microscopic Evaluation

#### 3.3.1. Qualitative Evaluation

**Figure 1:**
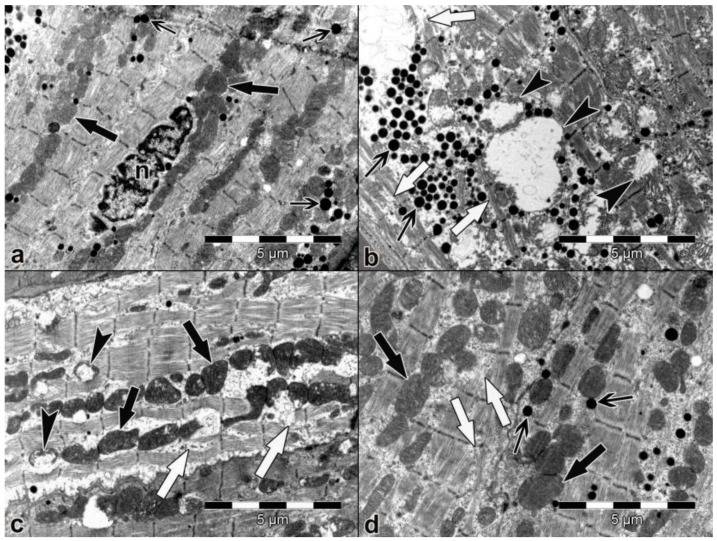
Electron micrograph of the atrium (×10,000). (**a**). Control group. Atrial muscle cells had normal morphological features. Normal mitochondrion (black thick arrow), atrial granule (thin arrow), and nucleus (n). (**b**). Saline group. Mitochondrial degeneration (arrow head), atrial granule (thin arrow), and degeneration in myofibrils (white arrow). (**c**). LEV100 group. Normal mitochondrion (black thick arrow), mitochondrial degeneration (arrow head), and degeneration in myofibrils (white arrow). (**d**). LEV200 group. Normal mitochondrion (black thick arrow), degeneration in myofibrils (white arrow), and atrial granule (thin arrow).

**Figure 2:**
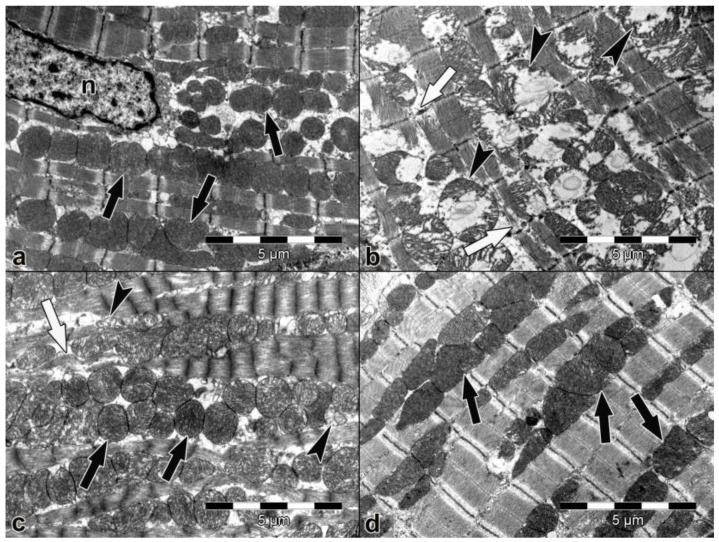
Electron micrograph of the ventricle (×10,000). (**a**) Control group. Ventricular muscle cells had normal morphological features. Normal mitochondrion (black thick arrow) and nucleus (n). (**b**) Saline group. Mitochondrial degeneration (arrow head) and degeneration in myofibrils (white arrow). (**c**) LEV100 group. Normal mitochondrion (black thick arrow), mitochondrial degeneration (arrow head), and degeneration in myofibrils (white arrow). (**d**) LEV200 group. Normal mitochondrion (black thick arrow).

Representative micrographs of atrial and ventricular heart muscle cells are shown in Figure 1 and Figure 2, respectively.

Atrial and ventricular muscle cells had normal morphological features in the control group. Nucleus, myofibrils, sarcomeric structures, mitochondria, and other sarcoplasmic organelles in both atrial and ventricular muscle cells were observed to be normal (Figure 1a and Figure 2a). In the saline group, there were prevalent degenerative alterations in some atrial and ventricular myocardial cells, and some myofibrils thinning and rupture were determined. Moreover, degenerative mitochondrial changes were more evident in this group (Figure 1b and Figure 2b). In the levetiracetam-treated groups (LEV100 and LEV200), although a great amount of atrial and ventricular myocardial cells had normal morphological features, mitochondrial and myofibril degenerations were detected in some heart muscle cells (Figure 1c,d and Figure 2c,d ).

#### 3.3.2. Semiquantitative Analysis of Mitochondrial Damage

##### Atrium

**Figure 3:**
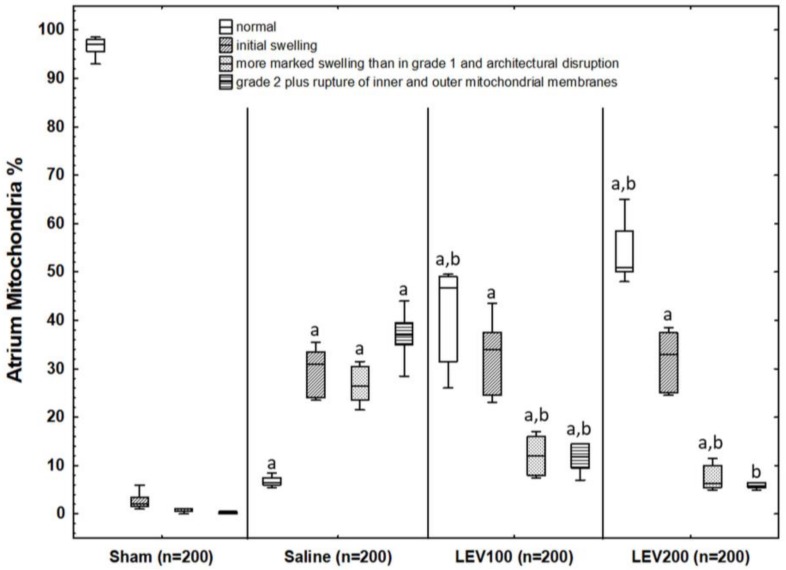
The percentage values of mitochondrial scores for atrium biopsies taken from the groups in the study. Error bars indicate the range of distribution; the box, the interquartile range; the horizontal line, median value. Values in parentheses are the number of mitochondria scored for each group. ^a^ *p* < 0.0001 vs. Control group, ^b^ *p* < 0.0001 vs. Saline group.

Figure 3 shows quantitative grading of mitochondrial damage in atrium for each group. As shown in Figure 3, biopsies taken from the control group indicated that the great number of mitochondria had almost no signs of injury (grade 0: 97% (94.88–98.13%), grade 1: 2% (1.38–4.13%), grade 2: 0.50% (0.37–1.00%), and grade 3: 0.25% (0–0.50%)), as expected. In the saline group, the number of mitochondria showing normal morphology was dramatically reduced (grade 0: 6.50% (5.87–7.75%), *p* < 0.0001) when compared to that of the control group. HI-induced mitochondrial damage in saline group was clearly observed via the number of mitochondria showing even higher scores in grade 1 (31% (23.87–34.00%)), grade 2 (26.50% (23.00–30.75%)) and grade 3 (37% (33.38–40.63%)) than those of the control group (*p* < 0.0001, respectively). These results showed that the HII can induce mitochondrial damage in neonatal rats’ atrium. Biopsies taken from the LEV100 and LEV200 groups had better mitochondrial morphology (grade 0: 46.75% (30.13–49.13%) in LEV100; grade 0: 51% (49.50–60.13%) in LEV200) than the saline group (*p* < 0.0001, respectively). The number of mitochondria in LEV100 and LEV200 graded as grade 2 (12% (7.87–16.25%) in LEV100, 6.25% (5.38–10.38%) in LEV200) and grade 3 (11.75% (8.88–17.63%) in LEV100; 5.75% (5.38–7.88%) in LEV200) were significantly lower than those of the saline group (*p* < 0.0001 for all comparisons). Scores in LEV100 and LEV200 with respect to the grade 1 (34% (24.12–39.00%) in LEV100; 33% (24.87–37.75%) in LEV200) were found to be similar with the saline group. Samples from the LEV200 group had better mitochondrial morphology (higher grade 0 score) than the LEV100 group, but no statistically significant differences were found among the groups. Grade 1, grade 2, and grade 3 scores were found similar between the LEV100 and LEV200 groups.

##### Ventricle

**Figure 4:**
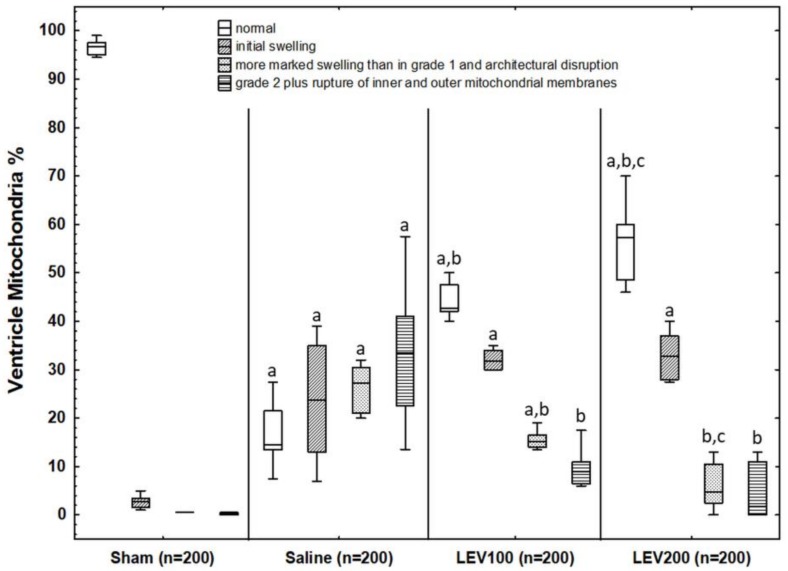
The percentage values of mitochondrial scores for ventricle biopsies taken from the groups in the study. Error bars indicate the range of distribution; the box, the interquartile range; the horizontal line, median value. Values in parentheses are the number of mitochondria scored for each group. ^a^ *p* < 0.001 vs. Control group, ^b^ *p* < 0.001 vs. Saline group, ^c^ *p* < 0.001 vs. LEV100 group.

Quantitative grading of mitochondrial damage for each group in ventricle is shown in Figure 4. Biopsies taken from the control group demonstrated that a great amount of mitochondria had nearly no signs of injury (grade 0: 96.75% (94.88–97.88%), grade 1: 2.75% (1.38–3.88%), grade 2: 0.50% (0.38–0.63%), and grade 3: 0% (0–0.50%)), as expected. As shown in Figure 4, the number of mitochondria demonstrating normal morphology was significantly reduced in the saline group (grade 0: 14.50% (12–23%), *p* < 0.0001) when compared to that of the control group. HI-induced ventricular mitochondrial damage in saline group was clearly observed via the number of mitochondria showing higher scores in grade 1 (23.75% (11.50–36.00%)), grade 2 (27.25% (20.75–30.88%)), and grade 3 (33.50% (20.25–45.13%)) than those of the control group (*p* < 0.0001, respectively). Biopsies taken from the LEV100 and LEV200 groups had better mitochondrial morphology (grade 0: 42.75% (41.50–48.13%) in LEV100; grade 0: 57.25% (47.87–62.50%) in LEV200) than the saline group (*p* < 0.0001, respectively). The number of mitochondria in the levetiracetam-treated groups graded as grade 2 (15.25% (13.88–17.13%) in LEV100; 4.75% (1.88–11.13%) in LEV200) and grade 3 (9.00% (6.38–12.63%) in LEV100; 1.75% (0–11.50%) in LEV200) were significantly lower than those of the saline group (*p* < 0.0001 for all comparisons). Grade 1 scores in LEV100 (31.75% (27.50–34.25%)) and LEV200 (32.75% (27.88–37.75%)) were observed to be similar with the saline group. LEV200 group had better mitochondrial morphology (higher grade 0 score) and less damaged mitochondria (lower grade 2 score) than the LEV100 group (*p* < 0.0001 respectively). Grade 1 and grade 3 scores were found to be similar among the groups.

## 4. Discussion

Newborns with asphyxia and hypoxic ischemic encephalopathy frequently suffer multiple organ failure as a result of the HII, including myocardial dysfunction [40,41]. Several clinical and experimental studies have been performed to evaluate the effects of HII on myocardial functions and cardiac mechanical features [31,42,43,44,45,46,47,48]. However, the results of these studies have pointed out the early effects of the HII on cardiac functions. The late effects of HII on myocardial functions and/or cardiac mechanical features are not clarified. In our study, the late effects of HII and the possible cardioprotective effects of LEV on rats in adulthood were investigated on the myocardial contraction and ultrastructure of the heart muscle. Thus, the early effects of HII on heart contractility were not taken into account.

In a pup HI model (right common carotid artery ligation at seven days after birth; 8% oxygen for 2 h) performed by Buyukakilli et al. [38], the late effects of HIBI were investigated on cardiac mechanical performance in rats in adulthood (16 week old) [38]. In this study, the authors revealed that the exposure to HI during the neonatal period caused a significant reduction in atrial contractile force [38]. In addition to that, ventricular contractile force in rats with HI was observed to be decreased in a nonsignificant manner [38]. However, in our study, no statistically significant differences were found between the control and the saline groups with regard to the atrial contractile force, the atrial contractile duration, and the ventricular contractile duration values of rats. However, compared to the control group, the rats in the saline group had significantly less ventricular contractility (i.e., ventricular contractile force), indicating that the exposure to HI during neonatal period may cause ventricular dysfunction in adult rats. Moreover, the reductions in HW, BW, and HW/BW among the mentioned groups could also be interpreted as a reflection of growth retardation and/or heart damage associated with HIBI in adult rats. On the other hand, the reductions in HW, BW, and HW/BW were observed to be attenuated by the both LEV administrations, indicating that the LEV might have both neuroprotective effects and cardioprotective effects on myocardial integrity against the HII. The decrease in ventricular contractility as a result of HII was observed to be prevented by 100 mg/kg LEV administration. However, unexpectedly, the rats in the LEV200 group had less ventricular contractile force, even from the saline group. The ventricular contractile durations of the levetiracetam-treated groups were found to be as long, or longer, than those of both control and the saline groups. When these results are taken into account, our findings showed that the ventricular dysfunction and heart damage resulting from exposure to HI during the neonatal period could be partly prevented by LEV administration in adult rats. 

Calcium (Ca^2+^) has a crucial role in the regulation of myocardial cell functions and is the link between the electrical signals that pervade the heart and the contraction of the cardiac muscle cells (i.e., cardiomyocytes) in the process known as cardiac excitation-contraction (EC) coupling [49]. During EC coupling, the presence of a complex network of sarcolemmal invaginations known as T-tubules plays an important role in the cytosolic Ca^2+^ regulation. As a result of the AP propagation carried out by the T-tubules, sarcolemma of each myocyte becomes depolarized, thereby causing an opening of L-type voltage-gated Ca^2+^ channels (VGCCs). Ca^2+^ ions flow via the VGCCs into a space known as “dyadic cleft” [49]. The accumulation of Ca^2+^ during an AP increases Ca^2+^ concentration within the cleft from ~100 nM to ~10 μM. This local Ca^2+^ influx is known as a “Ca^2+^ sparklet”. Ca^2+^ sparklets are inadequate to cause a significant contraction but are sufficient to induce opening of the type 2 ryanodine receptor (RyR2), which is responsible for Ca^2+^ release from the SR to the cytosol, via a process known as “Ca^2+^-induced Ca^2+^ release (CICR)” [49,50]. The activation of a cluster of RyR2s, and consequent Ca^2+^ release from the SR, produces a Ca^2+^ release signal known as a “Ca^2+^ spark.” With the activation of RyR2 and the release of Ca^2+^ from different adjacent source of RyR2s and VGCCs increases the Ca^2+^ concentration to >100 μM in the dyadic cleft [49]. Ca^2+^ ions diffuse out of the cleft to engage the contractile machinery. Ca^2+^ diffusion, and their ensuing spatial and temporal summation, gives rise to a global Ca^2+^ increase of 500 nM to ~1 μM. Ca^2+^ binding component of the contractile myofilaments, troponin C, is sensitive to that range of Ca^2+^ concentration thereby causes the AP-mediated Ca^2+^ transient and contraction [49,50]. As mentioned above, it is clear that any defect in Ca^2+^ signaling would impair cardiac contractility [49,50]. On the other hand, besides the functions in cellular energy production, mitochondria are also known to be implicated in intracellular Ca^2+^ signaling, cell metabolism, and cell survival by regulating cytosolic Ca^2+^ concentration [47]. Since the heart relies almost solely on the mitochondria for energy, contraction, and ion transport (H^+^, K^+^ and Ca^2+^), it makes sense that any defects in mitochondrial functions would also substantially affect the heart [38,51,52,53]. Owing to the fact that the structural organization of the cardiomyocytes, the ultrastructure of the mitochondria, and the local and global Ca^2+^ transients obviously play a vital role in Ca^2+^ regulation and cardiac contractility, this makes them the main targets of cardiotoxicity [49,50,51,52,53,54].

The ultrastructural and mitochondrial findings of our study indicated that the HII caused considerable ultrastructural degenerations in a large amount of ventricular myocytes (degenerated and fragmented myofibrils) and mitochondria (separated cristae, decreased matrix density, disrupted architectural integrity, ruptured inner and outer mitochondrial membranes). These alterations may be the reason for the HII-related reduced ventricular contractile force seen in the saline group. However, unlike the atrial mechanogram findings, significant degenerative alterations have been detected between the control and the saline groups with regard to the atrial ultrastructure and mitochondria. Presumably, in spite of HII, the presence of normal contracting atrium areas caused atrial contractility to continue normally. Some critical ultrastructural differences between the ventricular and the atrial myocytes that substantially affect the spatial features of the Ca^2+^ signals may cause this situation to happen. Unlike the ventricular myocytes, atrial cells have a less-developed T-tubule system (known as Z-tubules formed by internal SR membrane) [55]. This means that the process of CICR in the ventricular EC coupling is much more effective than that of the atrium because the T-tubules provide that Ca^2+^ is released in close proximity to all sarcomeres, regardless of how deep they lie within the cell [56]. T-tubules promote the synchronous and efficient activation of the cell. In this way, the ventricular myocytes show homogeneous responses as a consequence of the simultaneous cascade of Ca^2+^ sparks throughout the cell [55]. Second, the atrial myocytes have two RyRs cluster populations: (i) junctional RyRs that are crucial in atrial EC coupling placed just beneath the sarcolemma, and (ii) non-junctional RyRs located deeper inside the cell. As the third, the ventricular and atrial localization pattern of the VGCCs is completely different from each other, which provide the atrial EC coupling to occur only at the cell periphery [49,55]. These differences indicate that the Ca^2+^ transient is less synchronous in atrial cells, and simultaneously recruited Ca^2+^ signals do not propagate substantially into the center of the cell [56]. If the disrupted atrial areas are mostly located in close proximity to the center of the cell, this may not cause a significant change in the atrial contractility, and by this way, atrial myocytes could continue their normal contraction process. Similar findings have been previously reported by Buyukakilli et al. [38] with regard to the qualitative and semiquantitative electron microscopic analysis and they showed that the HII caused significant ultrastructural degenerations in both ventricular/atrial myocytes and ventricular/atrial mitochondria in rats with HII [38]. On the other hand, rats treated with 100 mg/kg and 200 mg/kg LEV (i.e., LEV100 and LEV200 groups) had better morphologic view and had significantly less ultrastructural degenerations in both ventricular/atrial myocytes (a less amount of degenerated and fragmented myofibrils) and mitochondria (higher grade 0 scores; lower grade 2 and 3 scores) than those of the rats with HII. These alterations indicated that the LEV administration may prevent the HII-induced reduction in ventricular contractile force and ultrastructural and mitochondrial damage in the atrium and ventricle of the adult rats.

Several researchers have revealed several mechanisms for the anti-seizure, the anti-epileptic, and the anti-convulsant effects of LEV [57,58]. Among these mechanisms, Oliviera et al. [29] have shown that the LEV could increase muscarinic acetylcholine receptor activity, especially muscarinic acetylcholine reseptor-2 (M_2_), in the pilocarpine-induced seizure model [29]. These receptors mediate various physiological functions including heart rate and force, contraction of smooth muscles, and the release of neurotransmitters. To date, five receptor subtypes (M_1_–M_5_) have been identified [30]. The M_2_ receptor is the dominant muscarinic receptor subtype in the heart [59]. The presence of the functional M_1_ and M_3_ receptors have been also identified and characterized in rat ventricular myocytes [60,61,62,63,64]. It is useful to recall that the muscarinic stimulation increases myofilament Ca^2+^ sensitivity in healthy rat myocardium with no apparent inotropic response and increased myofilament Ca^2+^ sensitivity promotes increased contractility in failing cardiac tissue [65]. Thus, muscarinic receptor activation could elicit inotropic responses in ventricular myocardium from rats with heart failure by increasing phosphorylation of myosin light chain [65]. According to this knowledge and in the view of the evidence coming from previous report [29], by this molecular mechanism, LEV may act as a receptor activator and could promote ventricular contractile force in rats treated with 100 mg/kg of LEV (i.e., LEV100 group). However, we are unable to explain why group LEV200 had less ventricular contractile force even from the saline group. On the other hand, researchers have found a possible interaction between the LEV and voltage-dependent K^+^ channels (K_V_) that play a vital role in determining the excitability of neurons [66]. K_V_ channels are responsible for regulating the resting potential, repolarizing membranes during action potentials, and setting action potential duration and frequency. Among these, delayed-rectifier K_V_ channels are ubiquitous in neurons [66,67]. In 2009, Huang et al. [65] reported that the LEV (30 µM) could suppress the amplitude of delayed rectifier K^+^ current in a concentration-dependent manner in differentiated NG108-15 neurons [66]. Delayed-rectifier K_V_ channels are also extensively present in cardiac muscle cells [68]. These channels and their currents (delayed rectifier outward potassium currents I_Kr_ (rapid) and I_Ks_ (slow)) play a dominant role in the phase 3 of myocardial action potential repolarization [69]. Reduced I_Ks_ mostly indicate long action potential duration. Moreover, the I_Ks_ blockade contributes significantly to drug-induced long QT syndrome [68,69,70,71]. Recently, Issa et al. [72] reported a case of a 24-year old female with a seizure disorder and previously undiagnosed long QT syndrome. In this report, they found that the QT interval of the patient was increased and developed into torsades de pointes after her LEV dose was increased (250 mg/twice a day to 1000 mg/twice a day of LEV). It is known that the QT interval in ECG displays the myocardial action potential duration [73]. The prolonged action potential may be the reason for the prolongation of atrial and ventricular contractile durations seen in the LEV treated groups. Further investigations should be carried out to discover the true extent of the cardiac effects of LEV.

## 5. Conclusions

On the basis of the results, it can be said that LEV is shown to possess protective features on the ventricular contractility and ultrastructural and mitochondrial damage in myocardium against neonatal HII-induced deteriorations in adulthood. It is, however, fair to state that owing to having possible negative effects on the atrial and ventricular contractile durations, which may cause serious cardiac problems such as long QT syndrome or torsades de pointes, the cardioprotective effects of LEV remain limited. To the best of our knowledge, our study is the first paper to show the effects of LEV on cardiac mechanical performance and ultrastructural properties of cardiac muscle in adulthood against neonatal HII. Exposure to HII in the neonatal period is known to contribute to the progression of multi-organ damage, including late-onset cardiovascular abnormalities. As LEV is broadly prescribed by clinicians for treating neonatal epileptic seizures because of its anti-epileptic activity against different seizure types with a good efficacy and safety profile, we think that it is important for clinicians to know its possible positive and negative effects on the mechanical performance and ultrastructural properties of myocardium in adulthood. Thus, more clinical trials on the subject are necessarily required.

## Figures and Tables

**Table 1:** Weight results of the groups.

Parameters	Control (*n* = 7)	Saline (*n* = 13)	LEV100 (*n* = 11)	LEV200 (*n* = 9)
Body Weight (BW; g)	262 (11)	240 (19) ^a‡^	238 (18) ^a‡^	248 (10)
Heart Weight (HW; g)	0.98 (0.04)	0.88 (0.10) ^a‡^	0.92 (0.07)	0.93 (0.09)
HW/BW ratio (mg/g)	3.73 (0.06)	3.67 (0.25)	3.85 (0.13)	3.76 (0.41)

^a^ vs. Control group. ^‡^ *p* < 0.05. Values are mean (SD).

**Table 2:** Mechanogram results of the groups.

Parameters	Control (*n* = 7)	Saline (*n* = 13)	LEV100 (*n* = 11)	LEV200 (*n* = 9)
Atrial contractile force (g)	0.334 (0.115)	0.351 (0.094)	0.350 (0.072)	0.308 (0.108)
Trial contractile duration (ms)	0.153 (0.034)	0.151 (0.020)	0.203 (0.029) ^a†,b†^	0.168 (0.023) ^c‡^
Ventricular contractile force (g)	2.75 (1.11)	1.34 (0.47) ^a†^	1.96 (0.85)	1.08 (0.19) ^a†,c‡^
Ventricular contractile duration (ms)	0.244 (0.043)	0.282 (0.078)	0.507 (0.157) ^a†,b†^	0.647 (0.166) ^a^*^,b^*

^a^ vs. Control group ^b^ vs. Saline group ^c^ vs. LEV100 group * *p* < 0.001 ^†^ *p* < 0.01 ^‡^ *p* < 0.05. Values are mean (SD).
